# Structural and functional changes of gut microbiota in ovariectomized rats and their correlations with altered bone mass

**DOI:** 10.18632/aging.103290

**Published:** 2020-06-02

**Authors:** Sicong Ma, Jinhong Qin, Yongqiang Hao, Ying Shi, Lingjie Fu

**Affiliations:** 1 Shanghai Key Laboratory of Orthopaedic Implants, Department of Orthopaedic Surgery, Shanghai Ninth People's Hospital, Shanghai Jiao Tong University School of Medicine, Shanghai, China; 2 Department of Microbiology and Immunology, Institutes of Medical Sciences, Shanghai Jiao Tong University School of Medicine, Shanghai, China; 3 Department of Orthopaedics, Shuguang Hospital Affiliated to Shanghai University of Traditional Chinese Medicine, Shanghai, China

**Keywords:** steroid deficiency-induced osteoporosis, gut microbiota, 16S rRNA sequencing, metagenomics, ovariectomized rats

## Abstract

As a critical factor involved in the maintenance of physiological homeostasis, the gut microbiota (GM) reportedly plays a key role in bone development. To date, the association between the GM and steroid deficiency-induced osteoporosis remains poorly understood. Forty female Sprague Dawley rats were divided into an ovariectomy (OVX) or control group. We performed 16S rRNA and metagenome sequencing, to compare diversity, taxonomic differences, and functional genes. The GM composition did not change in the control group and the number of operational taxonomic units increased significantly following ovariectomy. Alpha diversity, determined by ACE estimator, CHAO estimator, the Shannon index, and the Simpson index showed an increasing trend after ovariectomy. Samples in the OVX group were well clustered both pre- and post-ovariectomy, as demonstrated by principal coordinate 1 (PC1) and PC2. Functional genes of GM, including those involved in synthesis and metabolism of carbohydrates and nucleotides, microbial structure, and heme, as well as hemin uptake and utilization, increased at the early stage of osteoporosis. We observed that Ruminococcus flavefaciens exhibited the greatest variation in abundance among the GM and this was also associated with osteoclastic indicators and the estrobolome. Specific changes in fecal microbiota are associated with the pathogenesis of steroid deficiency-induced osteoporosis.

## INTRODUCTION

Osteoporosis is a systemic skeletal disease characterized by reduced bone mass and destruction of the bone tissue microstructure, with increased bone fragility and risk of fracture [1]. The pain, deformity, and death caused by osteoporotic fractures seriously affects the health of the elderly population [2]. The incidence of osteoporosis is up to 21% in women aged 50-84 and is approximately 6% in elderly men [3]. Apart from supplementation with calcium and vitamin D, the treatment for osteoporosis includes a variety of drugs with different mechanisms of actions; such drugs include bisphosphonates, selective estrogen receptor modulators, teriparatide, and denosumab [3]. However, adverse events may occur during treatment, including mandibular osteonecrosis, nephrotoxicity, and increased tumor risk [4]. Therefore, novel therapeutic targets to reverse osteoporosis-related bone loss are urgently required.

The gut microbiota (GM) plays a key role in a number of systemic disorders such as intestinal tumors, diabetes, non-alcoholic fatty liver, skin infections, and cardiovascular diseases [5–9]. Sjogren et al [10] found that germ-free (GF) mice exhibited increased bone mass with reduced number of osteoclasts compared to conventionally raised mice. Similarly, Li et al found that the GM was central in sex steroid deficiency-induced trabecular bone loss due to increased gut permeability, expansion of Th17 cells, and upregulation of osteoclastic cytokines [11]. Furthermore, administration of antibiotics in young mice increased adiposity and hormone levels because of the substantial taxonomic changes in the GM [12]. These findings suggest that the GM is also involved in maintaining bone mass. Therefore, thorough understanding of the changes in GM during steroid deficiency-induced osteoporosis may be beneficial for the prevention and treatment of postmenopausal osteoporosis.

The GM impacts metabolic homeostasis mainly by secretion of metabolites and modulation of the host immune systems. Short chain fatty acids (SCFAs) secreted by the GM may induce an increase in the transcription of calcium binding proteins in human and murine Caco-2 cells [13, 14]. Butyric acid may regulate intestinal regulatory T cell proliferation and enhance osteoclast differentiation [15]. In addition, the GM also maintains bone homeostasis by regulating calcium absorption-related proteins and modulating tight junction proteins [11, 16, 17]. GM-derived lipopolysaccharide (LPS) and muramyl dipeptide (MDP) indirectly affect osteoclast proliferation and differentiation by inducing an inflammatory response [18, 19].

To date, the relationship between the changes in GM composition and steroid deficiency-induced osteoporosis have not been fully determined [20]. In the current study, we performed 16S rRNA and metagenome sequencing to investigate the thorough structural and functional GM changes in an ovariectomized (OVX) rat model and determine the correlation between GM and steroid deficiency-induced osteoporosis.

## RESULTS

### Ovariectomy induces significant structural changes in the GM community

**Figure 1:**
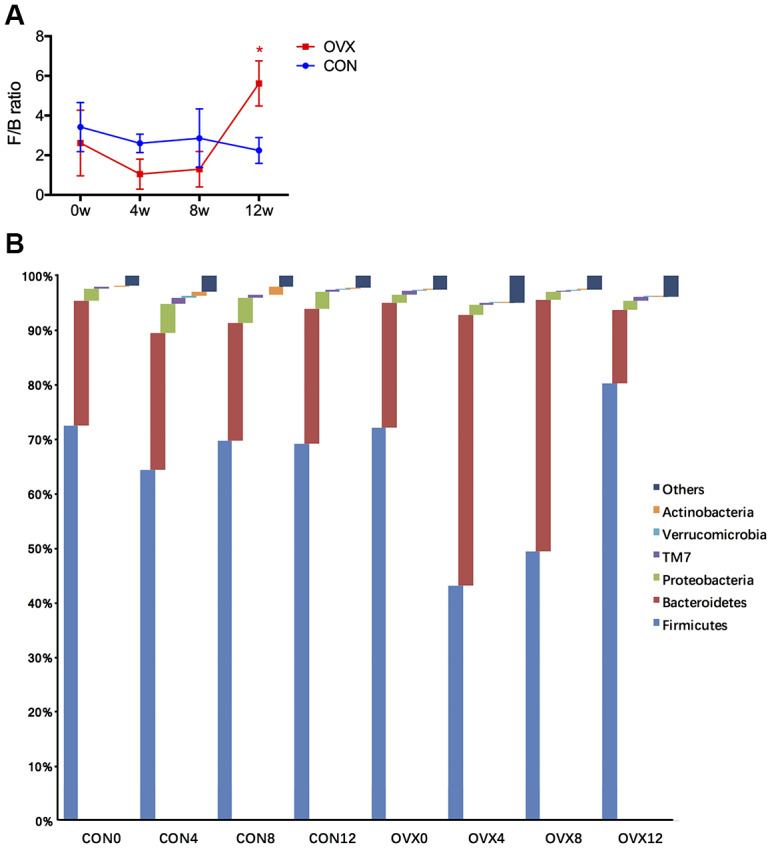
**Gut microbiota taxonomic composition in the CON and the OVX groups.** Firmicutes/Bacteroidetes ratio (F/B ratio) at different weeks following surgery (**A**). Annotation of phylum level for the six most abundant species of both groups (**B**).

The Firmicutes/Bacteroidetes ratio (F/B ratio) in the control group remained consistent during the whole study, while the F/B ratio in the OVX group at 12 weeks post-operation (OVX12) increased significantly compared with the F/B ratio before ovariectomy (OVX0) (P<0.05, Figure 1A). The GM remained steady in the CON group from CON0 to CON12 and the F/B ratios at the different time points in the CON group are similar to those in the OVX group at OVX0 (Figure 1B). These data suggest that the GM in the OVX group before ovariectomy (OVX0) is comparable to the GMs in the control group.

**Figure 2:**
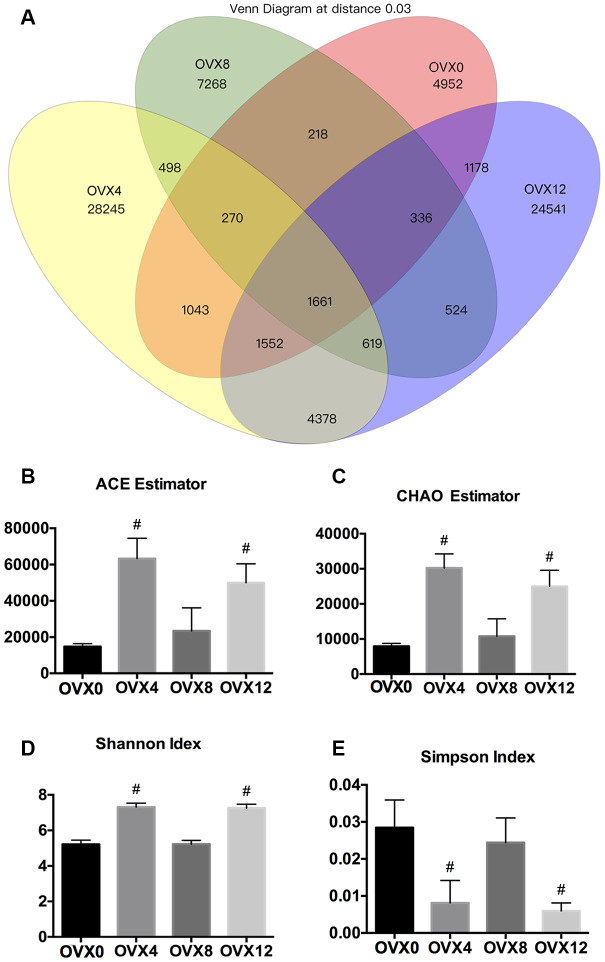
**Venn diagram and alpha diversity assessment of samples following ovariectomy.** Number of common and specific OTUs of samples with OTU=0.03 (**A**). Community richness assessment including the ACE estimator (**B**) and the Chao estimator (**C**); community diversity assessment including the Shannon index (**D**) and the Simpson index (**E**). Values were calculated using one-way ANOVA, #p < 0.01.

A Venn diagram was constructed to visualize the number of common and unique operational taxonomic units (OTUs). We observed that the number of unique OTUs (28245, 7268, and 24541 OTUs at OVX4, OVX8, and OVX12, respectively) increased significantly following ovariectomy while the lowest abundance of unique OTUs was detected at OVX0 (4952 OTUs) (Figure 2A). Next, alpha diversity was determined by ACE estimator, CHAO estimator, and Shannon index, identifying an increasing trend that suggested greater alpha diversity in the GM following ovariectomy (Figure 2B–2D). Furthermore, the gradually decreasing Simpson index also indicated greater alpha diversity in the OVX group (Figure 2E).

**Figure 3:**
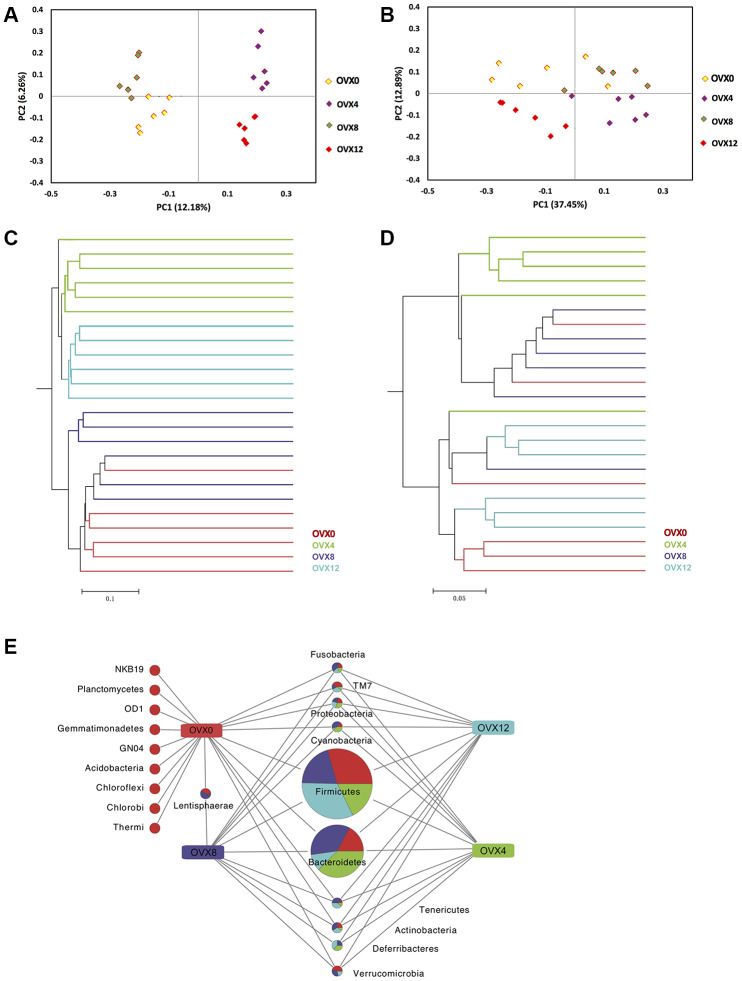
**Beta diversity assessment and ecological difference analyses after ovariectomy.** Principal coordinate analysis of 16S sequences using unweighted (**A**) and weighted (**B**) UniFrac showed distinct separation at different weeks following ovariectomy. Unweighted (**C**) and weighted (**D**) UniFrac hierarchical clustering trees also showed different GM composition. The common and characteristic phylum was obtained at OTU=0.03 (**E**).

To determine beta diversity following ovariectomy, unweighted and weighted UniFrac principal coordinate analyses (PCoA) were generated to visualize the ecological distance. The closer the points the better the samples clustered. As shown by the unweighted UniFrac PCoA in Figure 3A, samples were well clustered both pre- and post-ovariectomy in principal coordinate 1 (PC1) (percent variation explained: 12.18%) and PC2 (6.26%). Furthermore, weighted UniFrac PCoA also confirmed these findings (Figure 3B). The samples at OVX0 were separated from those at OVX12 in PC2 (12.89%). Meanwhile, when introducing PC1 (37.45%), the samples from different time points were separated well, too (Figure 3B). Unifrac distance algorithm is another method to determine the beta diversity by hierarchical clustering of distance matrices. Unweighted and weighted UniFrac trees clustered and distinguished well (Figure 3C, 3D). These data suggest that the samples collected from each time point in the OVX group were homogenous enough for subsequent sequencing analysis. More importantly, microbes such as Acidobacteria, Chloroflexi, and Thermi were enriched at OVX0 compared with those after ovariectomy (Figure 3E).

### Taxonomic differences between pre- and post-ovariectomy

**Figure 4:**
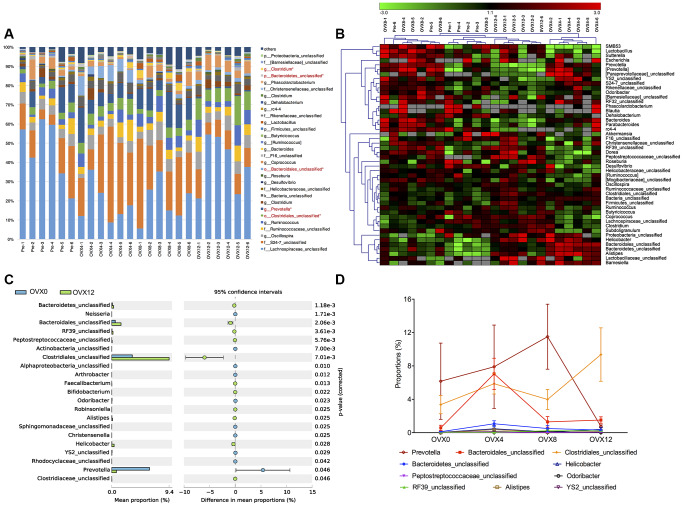
**Annotation of genus level for the thirty most abundant species determined by 16S rRNA sequencing following ovariectomy.** Microbiota in red font shows significant differences between OVX0 and OVX12 (**A**). Heat map of the microbial communities of samples were analyzed in genus level, OUT=0.03 (**B**). The abundant differences of genus comparison between pre- and post-ovariectomy (**C**). Species abundance changes of the ten most abundant genus following ovariectomy with significant differences (**D**). Values were analyzed using the Kruskal-Wallis H test with Benjamini-Hochberg FDR multiple test correction, *q<0.01.

GM taxonomy is of great importance for investigating the underlying mechanisms in metabolic diseases and correlating special species with pathogenesis. The Lachnospiraceae, Prevotella, Coprococcus, Ruminococcaceae and Clostridiales contents were more than 5% in pre- and post-ovariectomized rats (Figure 4A). In addition, Clostridium and Clostridiales of Firmicutes phyla increased significantly at OVX12, with Prevotella of Bacteroidetes phyla decreasing significantly at OVX12 compared with OVX0 (Figure 4A). The GM displayed the same trends after ovariectomy, as shown by the heat map analysis (Figure 4B). In order to determine detailed taxonomic differences, we further compared the abundances of microbes at OVX12 with those at OVX0. The abundances of Bactroidales and Clostridiales sub-groups increased significantly at OVX12 (Figure 4C). Interestingly, the abundance of Prevotella increased after ovariectomy until OVX8 and then decreased until OVX12 (Figure 4D). These species-specific changes may highlight candidates for the pathogenesis of steroid deficiency-induced osteoporosis. Ruminococcus flavefaciens was found to have the greatest variation of abundance in the clostridiales subgroup, which was also related to the estrobolome, an aggregate of enteric microbial genes (Supplementary Figure 1).

The gene sets named “estrobolome” is the aggregate of microbial functional genes which could produce estrogen metabolizing enzyme including β-glucuronidase and β-glucosidases [21]. Since the estrobolome is able to metabolize estrogens, decline of GM from estrobolome gene annotation may result in estrogen deficiency and lead to bone loss [22]. In the OVX group, Clostridium and Roseburia genus showed a increasing trend after ovariectomy (Supplementary Figure 2).

### Functional gene analyses of gut microbiome after ovariectomy

**Figure 5:**
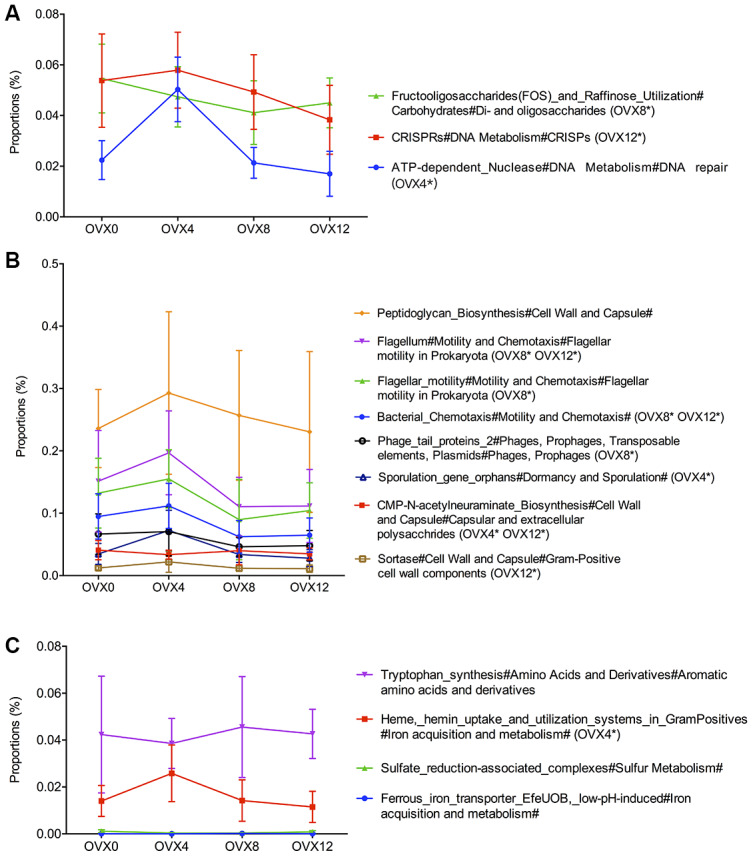
**Functional gene analyses of the gut microbiota after ovariectomy.** Functions related to synthesis and metabolism of carbohydrates and nucleotides (**A**), functions including microbial structure (**B**), and functions of synthesis and metabolism of small molecule organics compared with OVX0 (**C**). Values were analyzed using the Kruskal-Wallis H test with Benjamini-Hochberg FDR multiple test correction, *q<0.01, n=6.

First, the genes involved in synthesis and metabolism of carbohydrates and nucleotides are shown in Figure 5A. ATP-dependent nuclease (OVX0: 0.022 ± 0.008% *vs.* OVX4: 0.050 ± 0.013%) and CRISPR reads increased significantly at OVX4 and then decreased gradually until OVX12 (0.017 ± 0.009%). Second, genes involved in fructooligosaccharides (FOS) and raffinose metabolism decreased gradually (OVX0: 0.055 ± 0.014% *vs.* OVX8: 0.041 ± 0.013%). The reads of genes related to microbial structure reached a peak at OVX4 and then decreased rapidly until OVX12 (Figure 5B). Last, reads of tryptophan synthesis maintained a stable level. However, reads of heme as well as hemin uptake and utilization increased in the first 4 weeks after ovariectomy (OVX0: 0.014 ± 0.006% *vs.* OVX4: 0.026 ± 0.012%) (Figure 5C).

### Dynamic changes in bone mass and intestinal structure following ovariectomy

As expected, BMD decreased gradually after ovariectomy. BMD in the distal femur at OVX12 (0.1576 ± 0.0015 g/cm^2^) was significantly lower than that at OVX0 (0.1731 ± 0.0027 g/cm^2^) (P<0.01) (Supplementary Figure 3A). After ovariectomy, the level of serum estradiol showed the same trend as BMD. In addition, the osteoclastic index of CTX increased gradually, while the osteoblastic index of P1NP decreased following OVX (P<0.05) (Supplementary Figure 3B–3D). Qualitative and quantitative micro-CT measurements in the distal femur also confirmed osteoporotic changes (Supplementary Figure 3E, 3F). More importantly, these results were utilized in the following redundancy analysis (RDA) with taxonomy to determine the most specific GM species related to osteoporosis.

We next detected microstructure changes in the intestinal mucosa, with results showing that villus height decreased gradually (Supplementary Figure 3G). Moreover, the expression of the tight junction proteins Claudin-1, Occludin, and ZO-1 decreased significantly after ovariectomy (Supplementary Figure 3H–3J).

### Specific GM changes are associated with bone loss in ovariectomized rats

**Figure 6:**
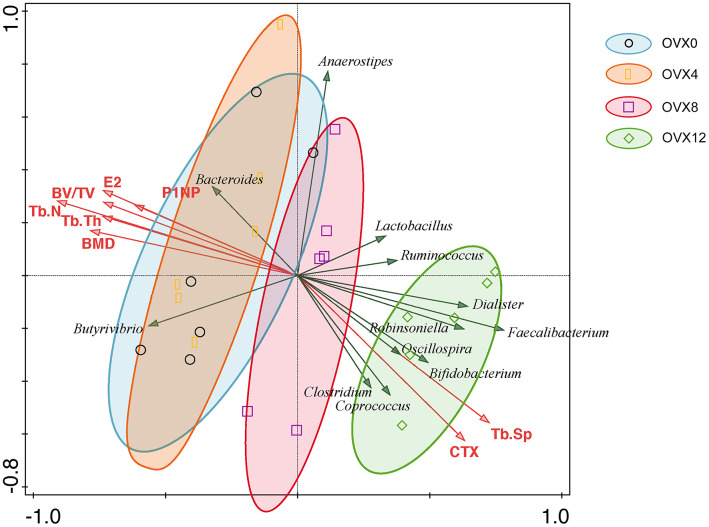
**RDA was performed to correlate bone mass with bone turnover markers with gut microbiota in ovariectomized rats.** The green arrows represent gut microbiota at the genus level and the red arrows represent biochemical indexes. The acute angle of arrows indicates the positive correlation between variables, and obtuse angles indicate negative correlations.

Redundancy analysis was used to correlate the GM with bone turnover parameters. The green arrows represented GM in genus level and the red arrows represented biochemical indexes. The acute angle of arrows indicated that the positive correlation between variables, and obtuse angle indicated negative correlation. As shown in Figure 6, Ruminococcus, Clostridium, Coprococcus, and Robinsoniella positively correlated with osteoclastic indicators and all samples of OVX12 were aligned with bone loss. However, Bacteroides and Butyrivibrio showed opposite patterns at OVX12 and were negatively correlated with loss of bone mass. On the whole, the GM showed obvious changes after ovariectomy and these specific microflora had significant positive correlation with the pathogenesis of steroid deficiency-induced osteoporosis.

## DISCUSSION

To the best of our knowledge, this is the first study to thoroughly characterize GM changes in an ovariectomized rat model. Our data suggest that steroid deficiency-induced osteoporosis was associated with GM dysbiosis characterized by an increase in alpha diversity and a significantly altered GM composition and function. Moreover, increased OTUs and intestinal dysfunction may play an important role in bone loss caused by estrogen deficiency after ovariectomy. In addition, Ruminococcus flavefaciens may be a pathological candidate for steroid deficiency-induced osteoporosis.

Firmicutes and Bacteroidetes are the main phylum of the GM that can be identified, accounting for 80% of the total microbiome [23]. The Firmicutes/Bacteroidetes ratio (F/B ratio) is an important and representative biomarker in several diseases such as diabetes, obesity, and hypertension [24–26]. In this study, we observed an increased F/B ratio after ovariectomy, suggesting that it can also be used as an effective biomarker for steroid deficiency-induced osteoporosis.

The GM seems to be a key factor for regulating bone mass by changing the structure of intestinal mucosa epithelium and mediating the activation of immune cells [18]. In the current study, shortened epithelial villi of the ileum and reduced mRNA expression of Claudin-1, Occludin, and ZO-1 after ovariectomy indicate a weakened intestinal barrier function. At the same time, the continuously increasing OTUs after ovariectomy established the altered gut microbiota composition in osteoporosis. Therefore, estrogen deficiency after ovariectomy may result in alterations to the GM, which then subsequently impair intestinal mucosal permeability and activate immune pathways, stimulating CD4^+^ T cells to increase osteoclasts, ultimately resulting in bone loss [27].

The composition of the GM determines the function of the microbiome in the intestine. Compared with the GM composition before ovariectomy, Clostridium, Robinsoniella, Coprococcus, and Dialister increased significantly at 12 weeks after ovariectomy. Robinsoniella and Dialister are associated with bone infection and bone volume loss, respectively [28–30]. In addition, the trend of DNA repair and CRISPR genes shows an increase during the first four weeks after ovariectomy, indicating that estrogen deficiency-related inflammatory reactions may lead to impairment of DNA repair [31]. Genes related to heme and hemin uptake and utilization systems in Gram Positive bacteria show a trend towards increased expression in osteoporotic rats. Crosstalk between the heme and iron-sulfur synthesizing pathways is essential for producing hemoglobin. In addition, iron–sulfur clusters are crucial for mitochondrial metabolism by microRNA-210, which is involved in the regulation of postmenopausal osteoporosis through promotion of VEGF expression and osteoblast differentiation [32–34].

One of the major advantages of metagenome sequencing is the ability to determine the species present in the GM. In the current study, Ruminococcus flavefaciens, which is also related to the estrobolome, was found to be the GM with the greatest variation in abundance. Ruminococcus flavefaciens could degrade cellulose with the products of SCFAs. However, high systemic concentrations of propionate and butyrate were toxic and may lead to adverse effects in the host, mostly arising from increased serum levels of SCFAs due to the enhanced “leak” of the gut barrier [35, 36]. These data imply that the increment of Ruminococcus flavefaciens after estrogen deficiency may produce excessive SCFAs in the intestine. Therefore, bone loss in osteoporosis may be the combined result of increased SCFAs and the compromised gut barrier function.

Prevotella levels are considered to be closely related with inflammatory bone loss [37, 38]. In the present study, Prevotella increased rapidly and maintained a high abundance in the early and middle stages of osteoporosis. In untreated rheumatoid arthritis (RA) patients, 16S sequencing of stool samples revealed an increased abundance of Prevotella, which is strongly correlated with bone loss [39]. Furthermore, literature shows that Prevotella functions through microbe associated molecular patterns (MAMPs) to activate various toll-like receptors (TLRs) and through principal immune cells to release inflammatory mediators and promote chronic inflammation [40]. These findings indicate that Prevotella may be a clinically important pathobiont for steroid deficiency-induced osteoporosis. An interesting finding related to Prevotella in this study is that this bacterium decreased eight weeks after ovariectomy. The possible explanation of this phenomenon may be due to a community shift and the inflammatory functional loss of Prevotella in the late stage of bone loss after ovariectomy.

One limitation of our study is that the results are descriptive and completely based on GM composition. Therefore, there is a distinct lack of mechanistic investigation (e.g. measurement of butyrate concentrations, GM transplantation, etc.). As such, all interpretations can only be considered speculative. The immune system plays a critical role in mediating GM-related effects on the regulation of bone mass. Cytokines and chemokines such as TNF-α, CCL-2, IL-10, SERT (Serotonin Transporter) and Tph1 may be possible mediators between the GM and steroid deficiency-induced osteoporosis [41, 42]. Therefore, further mechanistic studies, including fecal microbiota transplantation, are needed to verify the causality of gut microbiota on steroid deficiency-induced osteoporosis.

## CONCLUSIONS

This study demonstrated the extensive GM changes following ovariectomy, characterized by increases in alpha diversity and significant alteration of GM composition and function. Ruminococcus flavefaciens may be the possible pathological cause of steroid deficiency-induced osteoporosis.

## MATERIALS AND METHODS

### Experimental design and samples collection

The present study was approved by the Ethical Committee of Experimental Animal Care of the Shanghai Ninth People's Hospital (SH9H-2019-A17-1). Forty female Sprague Dawley (SD) rats (250 ± 20.0 g), aged 3 months, were purchased from Slac Laboratory Animal Company (Slac., China, SCXK2012-0002). Rats were housed two per cage with a 12-h light-dark cycle and allowed free access to water and pelleted rodent diet. Rats were randomly divided into two groups: the OVX and control (CON) groups. Bilateral ovariectomy was performed on the OVX group, following general anesthesia, and the same amount of adipose tissue was taken from the CON group [43]. Animals were sacrificed at 4, 8, or 12 weeks post-operation. Fecal samples (280 ± 20 mg) were acquired by direct collection methods from each rat in both groups. These GM samples were frozen immediately and stored at −80 °C for subsequent sequencing.

### Extraction and processing of microbial genomic DNA

Microbial genomic DNA was extracted from fecal samples using QIAamp DNA Stool Mini Kit (QIAGEN, USA). The primers 338F and 806R were used to amplify the V3-4 region of 16S rRNA genes. The thermocycling conditions for amplification included 20 cycles of 45 s at 95 °C, 30 s at 55 °C, and 30 s at 72 °C. Pyrosequencing was performed on an Illumina MiSeq instrument. Mothur (version 1.39.5) was used to assemble the paired FASTQ files [44].

Microbial DNA for metagenomic sequencing was fragmented into 400 bp reads and constructed by NEXTflex™ DNA Sequencing Kit compatible with the Biomek® FXp (Bio Scientific, USA). Then, we generated paired-end reads on Illumina HiseqTM2500 after cluster generation. Raw FASTQ files were filtered using FASTX-Toolkit. High-quality reads were spliced and assembled to obtain contigs with Mothur [44]. Scaffolds were constructed according to the known sequence of contigs. The main splicing parameter Kmer value was set to 55-85, and scaffolds of more than 500 bp were counted.

### Bioinformatics

High quality DNA sequences were grouped into operational taxonomic units (OTUs) and compared with the SILVA reference database (V128) at 97% similarity [45]. The minimum sample size was considered as the criteria of data normalization. Community richness and diversity analyses (ACE, Chao, Shannon, and Simpson index) were performed using Mothur. To visually study the similarity or difference of data, principal coordinate analysis (PCoA) was performed by calculating ecological distance to determine the eigenvalues and eigenvectors between samples. The thetaYC algorithm was used to calculate the similarity of community structure of each sample at OTU=0.03. The similarities and differences between multiple samples were described and compared using tree branch structures. Heat map and clustering relationship diagrams of samples were constructed according to the classification information of samples. Taxonomy was assigned using the online software RDP classifier (80% threshold) based on the Ribosomal Database Project [46, 47].

For gene prediction, the program Prodigal v2.60 was used to predict metagenome content from genes. Taxonomic characterization (phylum, class, order, family, genus, and species) was annotated based on previous results using the software Magen [48]. Bowtie2 v2.2.5 was used to compare clean reads with genes [49]. SAMtools v0.1.19 was used to calculate the read abundance of each gene in each sample [50]. The MARS (MA-plot-based method with Random Sampling) model in DEGseq was used to calculate differences in the expression abundance of each gene. Fold changes >1 and false discovery rate <0.001 were classified as significantly different. Diamond v0.9.1.102 was used to conduct BLASTP homology alignment between gene sequences and the SEED database. Functional annotation contents of genes were obtained with statistically significant E value <10^-10^. β-glucuronidases and β-glucosidases were considered as genes of the estrobolome [22]. All microflora in genus level sequenced by 16S rRNA in the current study was annotated based on “estrobolome”, and the obtained species was considered as estrobolome-associated bacteria. Redundancy analysis (RDA) was performed using the Vegan package in R.

### Dual energy X-ray measurement

Bone mineral density (BMD) of animals was determined by dual energy X-ray absorptiometry (DXA, Hologic Discovery A, USA).

### Micro-CT scanning

Distal femurs were scanned with a high resolution micro-CT 81system (Scanco Medical AG, Bruettiselien, Switzerland). The spatial resolution was 10 μm. Beam strength was set at 70 peak kV and 114 μA. Cubic voxels with a side length of 10 μm were used to represent measured objects [43]. A volume of interest (VOI) with 2-3 mm length of femoral trabecular region was selected (sigma=1.2, support=2, threshold=180). Histomorphometric parameters were computed by Scanco Medical software.

### ELISA assays

Serum estradiol, P1NP and CTX were measured using ELISA assay kits (XLCC, Shanghai YBL Biomedical Technologies, China) according to the manufacturer’s protocol.

### Intestinal H&E staining

For histological analyses, the rat intestine was cut longitudinally and then stained by H&E. Morphological characteristics were examined under light microscopy (Olympus, Japan).

### Real-time PCR

The mRNA of Claudin-1, Occludin, and ZO-1 were determined by real-time PCR. Real-time PCR was performed with TB Green Premix Ex Taq (Takara Bio Inc., Japan) using the ABI 7500 system (Applied Biosystems, USA). The primer sequences are shown in Supplementary Table 1.

### Statistical analyses

The statistical significance of bone mass and biochemical indexes was calculated using one-way analysis of variance (ANOVA) in GraphPad Prism (GraphPad Software Inc., USA, version 6.0). All data are expressed as mean values ± standard deviation (SD). Statistical significance was indicated as follows: *p < 0.05; #p < 0.01. Comparisons of abundance values for taxonomy and functional gene were analyzed using the Kruskal-Wallis H test with Benjamini-Hochberg FDR multiple test correction by STAMP version 2.1.3. Values with q<0.01 were considered statistically significant.

### Ethics approval

The study received ethics approval from the Ethics Committee of Shanghai Ninth People’s Hospital (SH9H-2019-A17-1).

## Supplementary Material

Supplementary Figures

Supplementary Table 1
