# Supplementary material for: Structural and functional changes of gut microbiota in ovariectomized rats and their correlations with altered bone mass
Source: Aging (Albany NY). 2020 Jun 2;12(11):10736–53. doi: 10.18632/aging.103290 (PMC7346027; doi:10.18632/aging.103290)
Supplement: Supplementary Figures [file aging-12-103290-s002..pdf]

SUPPLEMENTARY FIGURES

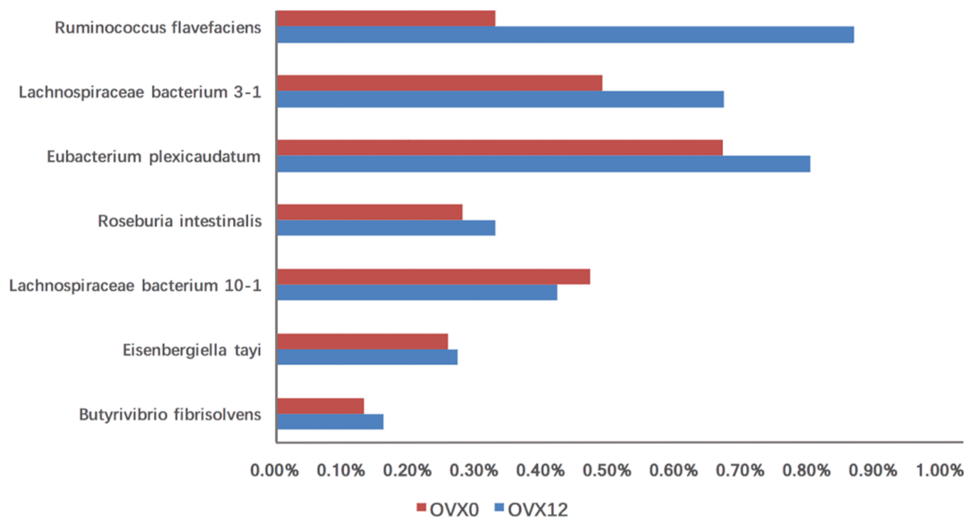

Supplementary Figure 1. Abundance with significant differences in sub-group of Clostridales at species level between pre- and twelve weeks post-ovariectomy, q<0.01.

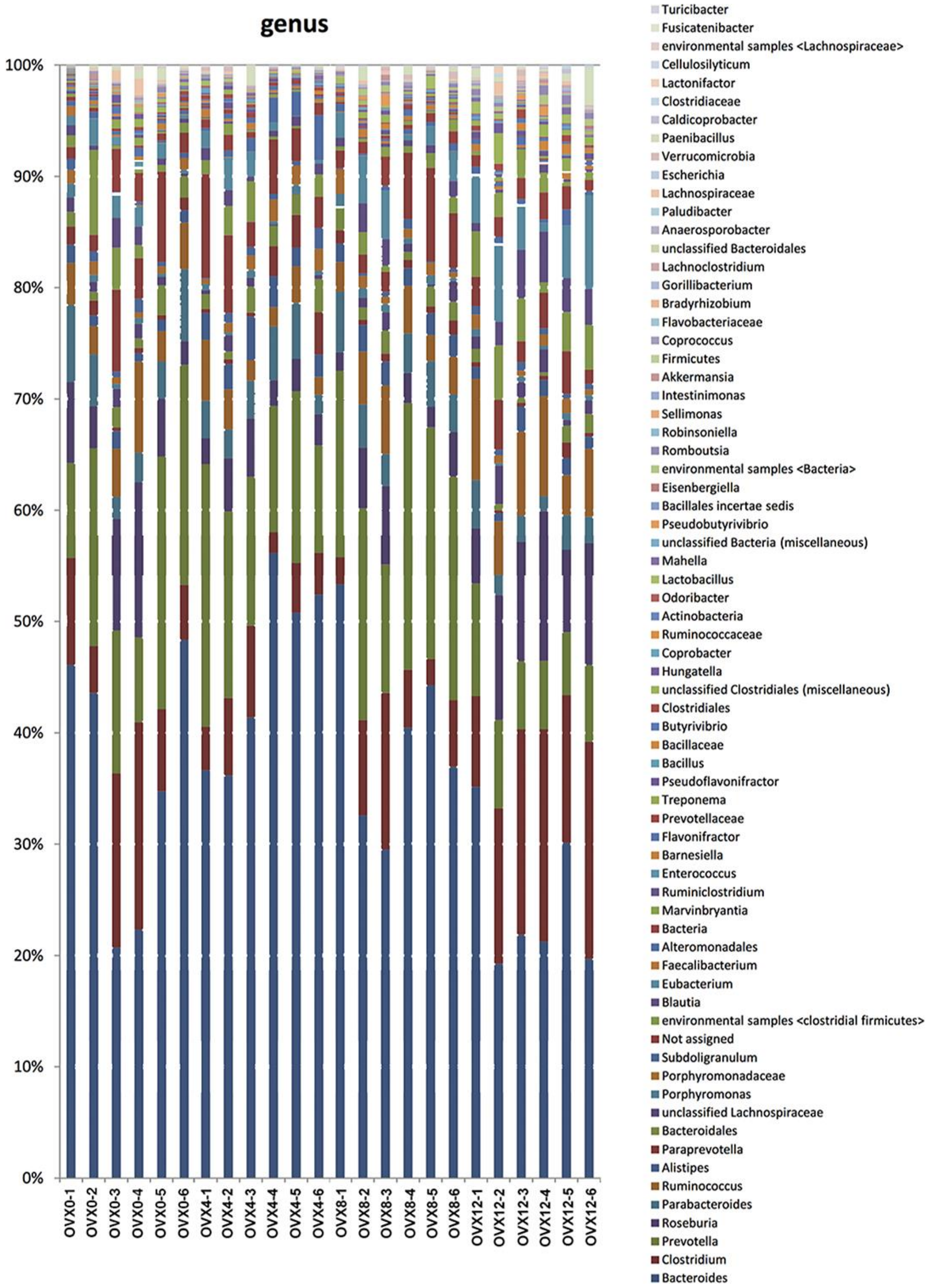

Supplementary Figure 2. The changes of gut microbiota related to the estrobolome.

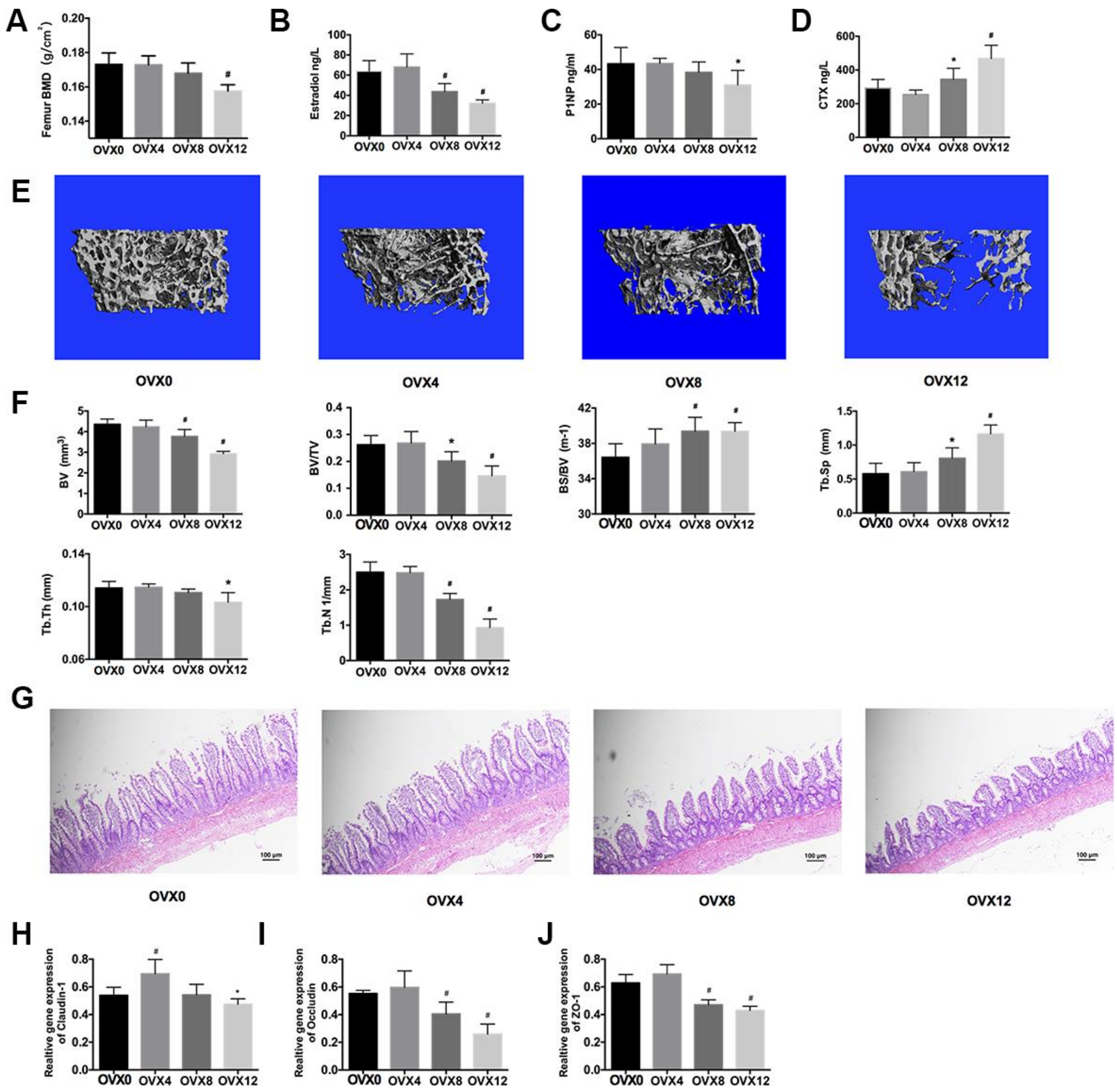

Supplementary Figure 3. Dynamic changes in biological data after ovariectomy. BMD (**A**), serum estradiol (**B**), P1NP (**C**), CTX (**D**), three-dimensional reconstruction of distal femur (**E**), and bone histomorphometric parameters (**F**): bone volume (BV), bone volume density (BV/TV), specific bone surface (BS/BV), trabecular spacing (Tb.Sp), trabecular thickness (Tb.Th), and trabecular number (Tb.N). Hematoxylin and eosin (H&E) staining of the intestine (100x) (**G**). mRNA expression of Claudin-1, Occludin and ZO-1 by real-time PCR (**H**–**J**). $^{*}P < 0.05$, $^{\#}P < 0.01$.
