# Supplementary material for: Structural and functional changes of gut microbiota in ovariectomized rats and their correlations with altered bone mass
Source: Aging (Albany NY). 2020 Jun 2;12(11):10736–53. doi: 10.18632/aging.103290 (PMC7346027; doi:10.18632/aging.103290)
Supplement: Supplementary Table 1 [file aging-12-103290-s001..pdf]

## SUPPLEMENTARY TABLE

**Supplementary Table 1. Primer sequences of tight junction proteins used for real-time PCR.**

| Name | | Primer sequence (5'-3') | Size (bp) | Accession No. |
|---|---|---|---|---|
| β-actin | F | CCTAAGGCCAACCGTGAAAA | 103 | NM_031144.2 |
| | R | CAGAGGCATACAGGGACAACAC | | |
| Claudin-1 | F | CATGAAGTGCATGAGGTGCTTAGAA | 107 | NM_031699.2 |
| | R | TGGCCACTAATGTCGCCAGA | | |
| Occludin | F | GTCTTGGGAGCCTTGACATCTTG | 174 | NM_031329.2 |
| | R | GCATTGGTCGAACGTGCATC | | |
| ZO-1 | F | CCATCTTTGGACCGATTGCTG | 123 | NM_001106266.1 |
| | R | TAATGCCCGAGCTCCGATG | | |
